# Supplementary material for: Glyphosate and AMPA in Human Urine of HBM4EU-Aligned Studies: Part B Adults
Source: Toxics. 2022 Sep 21;10(10):552. doi: 10.3390/toxics10100552 (PMC9612135; doi:10.3390/toxics10100552)
Supplement: Supplementary file 1 [file toxics-10-00552-s001.zip › toxics-1931303-supplementary.pdf]

Supplementary data

Table S1. Logistic binary regression for urinary concentrations of glyphosate (Gly) by some potential exposure determinants, subgroups of the participants stratified for individual characteristics, dietary preferences, exposure relevant behavior and sociodemographic information.

**Determinant**		**UBA ESB (Germany)**			**Swiss HBM4EU study (Switzerland)**			**DIET-HBM (Iceland)**			**ESTEBAN (France)**			**Combined data***		
		**N**	**Beta**	**p value**	**N**	**Beta**	**p value**	**N**	**Beta**	**p value**	**N**	**Beta**	**p value**	**N**	**Beta**	**p value**
Sex	Female	125	0		137			107	0		94			463	0	
	Male	124	0.88	**0.009**	162	-0.37	0.275	88	-0.29	0.408	74	-0.11	0.795	448	0.06	0.714
Age in years		249	0.06	0.421	299	-0.06	**0.060**	195	0.05	**0.090**	168	-0.06	**0.159**	911	-0.01	0.583
Nuts 1		All same category			All same category			All same category			168		0.788	911		0.002
Nuts 2		All same category			All same category			All same category			168		0.993	911		0.096
Nuts 3		All same category			299		1	195		0.362	168		1	911		1
DEGURBA	Densely populated area (cities)	All same category			273	0		146	0		76	0		744	0	
	Intermediate density area (towns or suburbs)				26	0.09	0.868	22	-0.28	0.622	47	-0.42	0.426	95	0.02	0.946
	Thinly populated area (rural area)							23	0.21	0.674	45	-0.02	0.959	68	0.21	0.564
Sampling season	Spring	All same category			147	0					36	0		432	0	
	Summer							65	0		38	-1.11	**0.067**	189	-0.62	0.048
	Fall				86	-0.14	0.716	104	0.11	0.767	61	-0.82	**0.100**	165	-0.42	0.255
	Winter				66	0.37	0.314	26	0.40	0.454	33	-1.95	**0.017**	125	-0.24	0.437
Use of pesticide outdoor	No				68	0					63	0		131	0	
	Yes				42	-0.06	0.918				23	0.81	0.211	65	0.29	0.513
Use of pesticides in the house	No				180	0					69	0		249	0	
	Yes				79	0.46	**0.194**				97	0.14	0.753	176	0.46	**0.110**
Frequency consumption of eggs**	0 = Never	3	0								3	0		6	0	

Determinant		UBA ESB (Germany)			Swiss HBM4EU study (Switzerland)			DIET-HBM (Iceland)			ESTEBAN (France)			Combined data*		
		**N**	**Beta**	**p value**	**N**	**Beta**	**p value**	**N**	**Beta**	**p value**	**N**	**Beta**	**p value**	**N**	**Beta**	**p value**
	Rarely: <1 time / month	19	-0.77	0.568	18	0					3	20.50	0.999	40	0.37	0.750
	Sometimes: <= 1 time / week but >= 1 time/month	89	-1.49	0.246	96	-0.42	0.503				111	19.65	0.999	296	-0.18	0.867
	Often: 2-3 times / week	90	-1.39	0.276	107	-0.62	0.335				42	19.17	0.999	239	-0.17	0.879
	Very Often: 4-6 times / week	34	-1.4	0.29	46	-0.17	0.806				4	20.10	0.999	84	0.05	0.965
	Everyday: >= 7 times / week	14	0.97	0.486	31	-2.32	0.051							45	0.26	0.826
Frequency consumption of fruit/vegetables***	0 = Never										2	0		2	0	
	Rarely: <1 time / month										3	-21.32	0.999	3	-22.28	0.999
	Sometimes: <= 1 time / week but >= 1 time/month	12	0					8	0		9	-0.76	0.650	29	-2.17	0.153
	Often: 2-3 times / week	29	0.06	0.949	2	0		41	-1.35	0.107	36	-1.67	0.281	108	-2.90	0.051
	Very Often: 4-6 times / week	62	0.33	0.682	3	-0.09	1	34	-1.58	0.070	46	-2.72	0.091	145	-3.13	0.034
	Everyday: >= 7 times / week	146	0.86	**0.27**	292	19.37	0.999	86	-1.05	0.185	67	-1.34	0.376	594	-2.46	0.095
Frequency consumption of cereals	0 = Never							19	0					19	0	
	Rarely: <1 time / month							8	-0.45	0.640				8	-0.63	0.520

Determinant		UBA ESB (Germany)			Swiss HBM4EU study (Switzerland)			DIET-HBM (Iceland)			ESTEBAN (France)			Combined data*		
		N	Beta	p value	N	Beta	p value	N	Beta	p value	N	Beta	p value	N	Beta	p value
	Sometimes: <= 1 time / week but >= 1 time/month							25	-0.34	0.620				25	-0.38	0.590
	Often: 2-3 times / week							53	-0.35	0.563				53	-0.47	0.449
	Very Often: 4-6 times / week							36	-0.65	0.333				36	-0.75	0.281
	Everyday: >= 7 times / week							29	-0.41	0.549				29	-0.51	0.457
Frequency consumption of local food	0 = Never															
	Rarely: <1 time / month															
	Sometimes: <= 1 time / week but >= 1 time/month															
	Often: 2-3 times / week															
	Very Often: 4-6 times / week															
	Everyday: >= 7 times / week															
Vegetarian	No	239	0					167	0		129	0		535	0	
	Yes	10	0.77	0.291				5	1.58	**0.121**	1	-19.70	1	16	0.855	**0.122**
Type of drinking water most consumed	Bottled water				34	0					43	0		77	0	
	Tap water				256	-0.62	0.166				101	-0.59	0.216	357	-0.74	**0.025**
	Ground water				5	-20.56	0.999				7	0.39	0.674	12	-1.31	0.238

Determinant		UBA ESB (Germany)			Swiss HBM4EU study (Switzerland)			DIET-HBM (Iceland)			ESTEBAN (France)			Combined data*		
		N	Beta	p value	N	Beta	p value	N	Beta	p value	N	Beta	p value	N	Beta	p value
	Other				3	0.49	0.710				15	0.30	0.665	18	0.31	0.962
Tap water source at home	Public				256	0								256	0	
	Private well				5	-20.01	0.999							5	-19.93	0.999
	Both public and private well															
Frequency of consumption of coffee****	0 = Never	43	0					43	0					86	0	
	Rarely: <1 time / month	16	-0.91	0.211				2	-21.62	0.999				18	-1.08	0.093
	Sometimes: <= 1 time / week but >= 1 time/month	23	-0.69	0.269										23	-0.80	0.169
	Often: 2-3 times / week	28	-0.62	0.282				15	-1.06	0.167				43	-0.82	0.067
	Very Often: 4-6 times / week	29	-0.47	0.403				11	0.15	0.840				40	-0.32	0.468
	Everyday: >= 7 times / week	110	-0.6	0.148				101	-0.78	0.061				211	-0.73	0.012
Facilities nearby home at time of sampling: farmland, orchard, or vinyard	No										1 filled in	0		1 filled in	0	
	Yes										15 filled in	19.02	1	15 filled in	19.83	1
Subject pregnant or occupationally exposed	Pregnant women															
	Occupationally exposed															
	Not pregnant or occcupationally exposed	All same category			All same category			All same category			All same category			All same category		

Determinant		UBA ESB (Germany)			Swiss HBM4EU study (Switzerland)			DIET-HBM (Iceland)			ESTEBAN (France)			Combined data*		
		N	Beta	p value	N	Beta	p value	N	Beta	p value	N	Beta	p value	N	Beta	p value
Labour status at time of sampling	Not employed/ Not actively working at the moment	All same category			58	0					31	0		338	0	
	Employed				239	-0.93	**0.007**	41 filled in employed			137	0.05	0.927	417	-0.73	**0.013**
Income by household*****	Low				61	0					23	0		84	0	
	Medium				159	-0.56	0.144				77	-0.23	0.692	236	-0.42	0.203
	High				54	-0.64	0.194				64	-0.66	0.293	118	-0.60	**0.140**
	Don't know or don't want to share										4	0.29	0.820	4	0.98	0.426
Highest education household	ISCED 0-2				3	0		11	0		3	0		17	0	
	ISCED 3-4				69	--2.09	0.117	58	0.57	0.541	45	19.78	0.999	172	0.22	0.740
	ISCED≥5	All same category			223	-2.72	**0.039**	123	1.01	0.269	120	19.70	0.999	715	0.20	0.759

NUTS: Nomenclature of territorial units for statistics

p values mentioned are pairwise p values except for age which was not categorical but continuous

Covariates forced into the model were creatinine, BMI, matrix (morning , spot urine, 24h urine); for combined studies also country was forced into the model.

OR= exp (Beta)

*: LOQ set at 0.1 µg/L

**: frequency of egg consumption was not significant for the UBA ESB study after recombination of categories

***: there was no significant trend in Gly concentrations for frequency of fruit&vegetable consumption for the combined studies after recombination of categories

****: there was no significant difference in Gly concentrations for frequency of coffee consumption for the combined studies between never drinking coffee an drinking coffee very often

*****: Country specific

Note that if a trend was observed in the data and the distribution of participants was unequal over different categories or the number of participants was small for certain categories, a combination of categories was performed. Here results are displayed as observed in the original harmonized questionnaire. Not all studied exposure determinants are listed in the table above; most relevant were chosen.

Table S2. Logistic regression for urinary concentrations of AMPA by some potential exposure determinants, subgroups of the participants stratified for individual characteristics, dietary preferences, exposure relevant behavior and sociodemographic information.

**Determinant**		**UBA ESB (Germany)**			**DIET-HBM (Iceland)**			**ESTEBAN (France)**			**Combined data***		
		**N**	**Beta**	**p value**	**N**	**Beta**	**p value**	**N**	**Beta**	**p value**	**N**	**Beta**	**p value**
Sex	Female	125	0		107			95	0		327	0	
	Male	124	0.44	**0.162**	88	-0.03	0.934	74	-0.06	0.889	286	0.121	0.540
Age in years		249	-0.01	0.931	195	0.03	0.321	168	0.06	0.880	613	0.01	0.747
Nuts 1		All same category			All same category			168		0.854	613		<0.001
Nuts 2		All same category			All same category			168		0.998	613		<0.001
Nuts 3		All same category			195		0.420	168		1	613		0.354
DEGURBA	Densely populated area (cities)	All same category			146	0		76	0		471	0	
	Intermediate density area (towns or suburbs)				22	-0.29	0.652	47	0.09	0.838	69	0.18	0.591
	Thinly populated area (rural area)				23	-0.58	0.380	46	0.73	0.156	69	-0.10	0.771
Sampling season	Spring	All same category						37	0		286	0	
	Summer				65	0		37	-0.72	0.224	102	-0.53	0.253
	Fall				104	-0.22	0.611	62	0.07	0.884	166	-0.10	0.809
	Winter				26	0.83	**0.135**	33	0.09	0.879	59	-0.04	0.928
Use of pesticide outdoor	No							63	0		63	0	
	Yes							25	0.535	0.431	25	0.512	0.402
Use of pesticides in the house	No							68	0		68	0	
	Yes							99	0.581	**0.160**	99	0.031	0.933
Frequency consumption of eggs**	0 = Never	3	0					3	0		6	0	
	Rarely: <1 time / month	19	19.49	0.999				3	-0.84	0.672	22	0.81	0.525

Determinant		UBA ESB (Germany)			DIET-HBM (Iceland)			ESTEBAN (France)			Combined data*		
		N	Beta	p value	N	Beta	p value	N	Beta	p value	N	Beta	p value
	Sometimes: <= 1 time / week but >= 1 time/month	89	19.46	0.999				113	-0.10	0.939	202	0.75	0.525
	Often: 2-3 times / week	90	19.55	0.999				41	0.26	0.843	131	0.75	0.525
	Very Often: 4-6 times / week	34	19.64	0.999				4	-1.69	0.370	38	0.85	0.489
	Everyday: >= 7 times / week	14	21.13	0.999							14	2.36	0.072
Frequency consumption of fruit/vegetables	0 = Never							2	0		2	0	
	Rarely: <1 time / month							3	-0.72	1	3	-21.27	0.999
	Sometimes: <= 1 time / week but >= 1 time/month	12	0		8	0		11	-19.34	0.999	31	-20.99	0.999
	Often: 2-3 times / week	29	-0.18	0.829	41	-1.42	0.115	35	-20.66	0.999	105	-21.70	0.999
	Very Often: 4-6 times / week	62	-0.59	0.448	34	-0.97	0.277	45	-20.29	0.999	141	-22.01	0.999
	Everyday: >= 7 times / week	146	-0.01	0.992	86	-0.92	0.262	68	-20.58	0.999	300	-21.53	0.999
Frequency consumption of cereals	0 = Never				19	0					19	0	
	Rarely: <1 time / month				8	-0.70	0.561				8	-0.70	0.561
	Sometimes: <= 1 time / week but >= 1 time/month				25	-0.27	0.718				25	-0.27	0.718

Determinant		UBA ESB (Germany)			DIET-HBM (Iceland)			ESTEBAN (France)			Combined data*		
		N	Beta	p value	N	Beta	p value	N	Beta	p value	N	Beta	p value
	Often: 2-3 times / week				53	-0.35	0.602				53	-0.35	0.602
	Very Often: 4-6 times / week				36	-0.23	0.749				36	-0.23	0.749
	Everyday : >= 7 times / week				29	-0.03	0.965				29	-0.03	0.965
Frequency consumption of local food	0 = Never												
	Rarely: <1 time / month												
	Sometimes: <= 1 time / week but >= 1 time/month												
	Often: 2-3 times / week												
	Very Often: 4-6 times / week												
	Everyday : >= 7 times / week												
Vegetarian	No	239	0		167	0		131	0		537	0	
	Yes	10	-0.09	0.912	5	2.04	**0.049**	1	20.46	1	16	0.41	0.484
Type of drinking water most consumed	Bottled water							44	0		44	0	
	Tap water							101	-0.43	0.373	101	-0.18	0.666
	Ground water							7	-1.06	0.314	7	0.61	0.538
	Other							15	-0.05	0.948	15	0.255	0.715
Tap water source at home	Public												
	Private well												

Determinant		UBA ESB (Germany)			DIET-HBM (Iceland)			ESTEBAN (France)			Combined data*		
		N	Beta	p value	N	Beta	p value	N	Beta	p value	N	Beta	p value
	Both public and private well												
Frequency of consumption of coffee	0 = Never	43	0		43	0					86	0	
	Rarely: <1 time / month	16	-1.14	**0.15**	2	0.67	0.652				18	-0.78	0.231
	Sometimes: <= 1 time / week but >= 1 time/month	23	-0.73	0.275							23	-0.80	0.198
	Often: 2-3 times / week	28	0.03	0.963	15	0.35	0.633				43	0.11	0.789
	Very Often: 4-6 times / week	29	-0.43	0.462	44	0.65	0.439				40	-0.18	0.699
	Everyday : >= 7 times / week	110	0.37	0.373	101	-0.14	0.762				211	0.17	0.580
Facilities nearby home at time of sampling: farmland, orchard, or vinyard	No							1 filled in	0		1 filled in	0	
	Yes							15 filled in	22.75	1	15 filled in	-20.54	1
Subject pregnant or occupationally exposed	Pregnant women												
	Occupationally exposed												
	Not pregnant or occcupationally exposed	All same category			All same category			All same category			All same category		
Labour status at time of sampling	Not employed/ Not actively working at the moment	All same category						30	0		279	0	

Determinant		UBA ESB (Germany)			DIET-HBM (Iceland)			ESTEBAN (France)			Combined data*		
		N	Beta	p value	N	Beta	p value	N	Beta	p value	N	Beta	p value
	Employed				41 filled in employed			139	-0.91	**0.134**	180	-0.05	0.909
Income by household**	Low							23	0		23	0	
	Medium							75	-0.16	0.789	75	-0.43	0.428
	High							67	0.65	0.299	67	-0.47	0.396
	Don't know or don't want to share							4	-1.08	0.407	4	-20.56	0.999
Highest education household	ISCED 0-2				11	0		3	0		14	0	
	ISCED 3-4				58	0.63	0.534	46	-17.98	0.999	104	1.04	0.242
	ISCED≥5	All same category			123	0.62	0.531	120	-18.71	0.999	492	1.29	0.138

NUTS: Nomenclature of territorial units for statistics

p values mentioned are pairwise p values except for age which was not categorical but continuous

Covariates forced into the model were creatinine, bmi, matrix (morning , spot urine, 24h urine); for combined studies also country was forced into the model.

OR= exp (Beta)

*: LOQ set at 0.1 µg/L

**: Country specific

Note that if a trend was observed in the data and the distribution of participants was unequal over different categories or the number of participants was small for certain categories, a combination of categories was performed. Here results are displayed as observed in the original harmonized questionnaire.

Not all studied exposure determinants are listed in the table above; most relevant were chosen.
